# Switching from imiglucerase to miglustat for the treatment of French patients with Gaucher disease type 1: a case series

**DOI:** 10.1186/s13256-015-0617-5

**Published:** 2015-06-23

**Authors:** Christine Serratrice, Laure Swiader, Jacques Serratrice

**Affiliations:** 1 Department of Internal Medicine, Foundation Hospital Saint Joseph, 26 Boulevard de Louvain, Marseille, 13008 France; 2 Department of Internal Medicine, CHU Timone, Marseille, France

**Keywords:** Gaucher disease, Imiglucerase, Miglustat

## Abstract

**Introduction:**

Gaucher disease is caused by a deficiency of the enzyme β-glucocerebrosidase. Treatment with enzyme replacement therapy has been available for the past two decades but, although effective, enzyme replacement therapy can be delivered only by intravenous infusion every other week. The oral substrate reduction therapy miglustat (Zavesca®) has been available in Europe since 2002 for the treatment of patients with mild or moderate Gaucher disease type 1 for whom enzyme replacement therapy is unsuitable or not a therapeutic option. There are few published real-world data on the use of miglustat as a maintenance therapy in Gaucher disease type 1 patients switched from previous enzyme replacement therapy. We report a case series of three patients who were switched from long-term enzyme replacement therapy to miglustat for various reasons.

**Case presentation:**

All three patients were Caucasian and had confirmed Gaucher disease type 1. An 80-year-old man requested a switch to oral miglustat therapy in preference to ongoing intravenous enzyme replacement therapy, a 57-year-old woman was commenced on miglustat due to a shortage of imiglucerase, and a 56-year-old woman was switched from previous enzyme replacement therapy due to allergic reactions to intravenous infusions. Hematological disease parameters were stable in each patient on previous enzyme replacement therapy. Two patients continue to be treated with miglustat, having shown good tolerability and stable core disease parameters for approximately 4 years. One patient, who was also stable during 7 years of therapy, eventually discontinued miglustat as a precaution because he developed peripheral neuropathy of as yet unknown origin.

**Conclusions:**

Overall, our experience indicates that miglustat can be used as maintenance therapy for Gaucher disease type 1 after initial enzyme replacement therapy, but the selection of patients to whom this approach should be applied should be made after careful consideration of all disease parameters.

## Introduction

Gaucher disease (GD) is an inherited lysosomal disorder caused by impaired activity of the enzyme, β-glucocerebrosidase, and accumulation of glucosylceramide in pathologic macrophages in various tissues including the liver, spleen and bone marrow [1]. GD type 1 (GD1) is the most common clinical variant, and is characterized by anemia, thrombocytopenia, hepatosplenomegaly and various skeletal manifestations [1, 2].

Enzyme replacement therapy (ERT) with imiglucerase (Cerezyme®; Genzyme Corp.) represented the only therapy for GD1 for more than a decade. ERT is well established as being effective in reducing the hematological, visceral and bone symptoms of GD1 [3, 4]. However, it comes with a substantial therapeutic burden due to the need for regular intravenous infusions.

Oral substrate reduction therapy (SRT) with miglustat (Zavesca®; Actelion Pharmaceuticals) was approved in the European Union, USA and various other countries for the treatment of adults with mild or moderate GD1 for whom ERT is unsuitable/not a therapeutic option based on data from several clinical trials [5–7]. Miglustat has become an alternative treatment strategy to ERT, with potential therapeutic effects on bone manifestations [8].

The switching of patients with GD1 from ERT to miglustat treatment was subject to intense focus during the global shortage of imiglucerase from 2009 to 2010 [9], but there were few published data on maintenance therapy with miglustat in patients with GD1 switched from previous ERT to draw upon at that time [10]. Since then, findings from a clinical non-inferiority trial assessing the effectiveness, safety and tolerability of miglustat as maintenance therapy in 42 patients with GD1 stabilized on previous ERT indicated satisfactory maintenance of core disease parameters [11]. Data from a cohort study with 115 patients treated with miglustat, 70% of whom previously received ERT, indicated a general stability of hematological parameters in patients who previously received ERT [12].

Overall, available reported data from patients switched from previous ERT suggest that responses to SRT can vary. However, there are few reports detailing issues related to clinical decision making in terms of (1) the reasons for switching to oral SRT, (2) patient factors that affect responses to SRT, and (3) discontinuation of SRT in cases of adverse events (whether drug related or not). The following report describes clinical experience with three different cases, where patients switched from imiglucerase to miglustat for a variety of reasons.

## Case presentation

### Patient 1

An 80-year-old man with noninsulin-dependent diabetes was diagnosed as having GD1 in 1992 (aged 59 years) following observed thrombocytopenia before prostate surgery. He had no current or previous bleeding at this point. Diagnosis was established based on β-glucosidase activity (0.28mkat/kg) and genetic testing (N370S/L444P mutant genotype). He had a platelet count of 50×10^9^/l, a spleen size of 16cm for longest axis and a normal liver size (measured by abdominal ultrasound); no plasma chitotriosidase levels were measured at the time.

He was started on ERT with imiglucerase 60IU/kg every 2 weeks in 2002. At that time he complained of bruising and epistaxis and had a platelet count of 25×10^9^/l, a hemoglobin concentration of 13g/dl, and spleen and liver sizes of 18cm and 16cm, respectively. Dual-energy absorptiometry (DEXA) indicated osteopenia based on femoral neck and lumbar spine T-scores (–2.2 and –1.6, respectively). All four core disease parameters stabilized after 3 years of ERT. His platelet count and hemoglobin concentration had increased to 94×10^9^/l and 15g/dl, respectively, and spleen and liver sizes had decreased to 13cm and 16cm, respectively. No bone manifestations were evident.

He requested a switch to oral SRT with miglustat in 2005 as he no longer wished to receive intravenous ERT infusions, and miglustat therapy was started in November that year at a dose of 100mg/day for 10 days, then 200mg/day for 10 days, and finally 300mg/day. No dietary alterations were applied. He lost 3kg in body weight during the initial 6 months of miglustat therapy, but did not complain of diarrhea or flatulence. He also experienced discrete limb tremor that did not affect his physical function. Findings from several separate electromyography (EMG) investigations were normal.

**Fig. 1:**
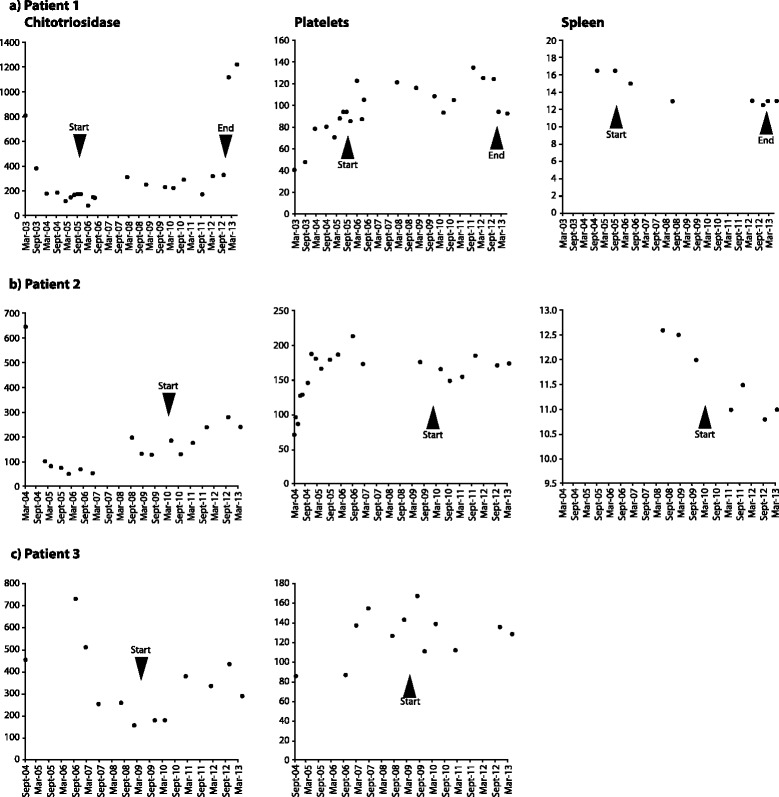
**a**–**c** Disease parameters in three patients switched from enzyme replacement therapy to miglustat. *Arrowheads* indicate start and/or end of miglustat therapy

In 2010, aged 77 years, he experienced a transient ischemic attack, with a repeat occurrence 1 year later. He made a full recovery in both instances, with no clinical sequelae. Follow-up monitoring, 7 years after he started miglustat therapy, showed that core hematological GD parameters were maintained (Fig. 1a). His hemoglobin concentration was 13.2g/dl, platelet count was 125×10^9^/l, and chitotriosidase activity was 320nmol/ml/hour (normal range <120nmol/ml/hour). His spleen size was 13cm, and liver size was 16cm. Whole-body magnetic resonance imaging (MRI) indicated an absence of bone lesions, and DEXA indicated normal femoral neck and lumbar spine T-scores at last follow up (–1.5 and –1.3, respectively).

He developed signs of peripheral neuropathy affecting his lower limbs at the age of 79 years (late 2012), and peripheral neuropathy was confirmed by EMG testing. Previous data have demonstrated that peripheral neuropathy can represent part of the natural history of GD [13]. In addition, because he had diabetes it was difficult to define if the neuropathy was due to diabetes, GD or miglustat. Nevertheless, it was decided to discontinue miglustat therapy and monitor him closely as a precaution. Two months later his platelet count had decreased to 56×10^9^/l, and there was a dramatic rise in his plasma chitotriosidase activity (from 333nmol/ml/hour to 1120nmol/ml/hour at last follow up), which stabilized after a further 4 months. He also gained 7kg in body weight after 3 months without therapy. He has not experienced bone pain and continues to be closely monitored with no treatment.

### Patient 2

A 57-year-old woman was diagnosed as having GD1 in 1983 at the age of 27 years due to thrombocytopenia and splenomegaly that were observed after a car accident. Her β-glucosidase activity was 0.5mkat/kg at diagnosis. Like Patient 1, she had a compound heterozygote mutant genotype (N370S/L444P). Prior to GD1-specific treatment she had a platelet count of 77×10^9^/l, a hemoglobin concentration of 12.3g/dl, and a spleen size of 18cm. There was no history of bone manifestations, bone scintigraphy findings were normal, and DEXA in lumbar spine and femoral neck indicated normal T-scores (–1.89 and –0.59 respectively).

Worsening thrombocytopenia (platelet count 61×10^9^/l), continuing splenomegaly and a raised plasma chitotriosidase activity (2329nmol/ml/hour) at follow up in 2002 prompted the commencement of ERT with imiglucerase, starting with a dose of 30IU/kg every 2 weeks, and continuing as such for 7 years up to 2009. At this point she was considered stable. Her platelet count was 226×10^9^/l, hemoglobin concentration was 12.3g/dl, spleen size was 11cm and plasma chitotriosidase was 105nmol/ml/hour. However, she had developed osteopenia, with a lumbar spine DEXA T-score of –1.8 (0 in femur) and an asymptomatic left diaphyseal infarction.

In July 2009, problems with the supply of imiglucerase led to an interruption in her imiglucerase therapy, and miglustat therapy was started approximately 1 year later, with progressive dose escalation and accompanied by a low-lactose, low-carbohydrate diet. Prior to miglustat therapy her platelet count was 168×10^9^/l, and her plasma chitotriosidase was 130nmol/ml/hour. After almost 2 years of miglustat therapy, her platelet count was maintained at 185×10^9^/l and remained approximately at this level until latest follow up (Fig. 1b). Chitotriosidase activity showed a modest increase (from 186nmol/ml/hour in mid-2010 to 280nmol/ml/hour approximately, in early 2012). No new bone manifestations occurred during miglustat therapy. Repeat DEXA measurements showed maintained lumbar spine bone mineral density (BMD; T-score –1.8), and a moderate improvement in femur BMD (T-score 0.3).

Overall, miglustat was well tolerated. She lost 2kg in body weight and experienced diarrhea in the first 2 months of therapy, but these resolved without medication by 6 months. She also experienced fatigue that progressed during the course of 2 years after the initiation of therapy, and which has still not completely resolved. Her neurological examination findings were normal.

### Patient 3

A 56-year-old woman was diagnosed as having GD1 at the age of 45 following 3 years of investigations for unexplained thrombocytopenia and asthenia. At diagnosis in 2002, her β-glucosidase activity was 0.5mkat/kg, and genetic analysis revealed a W312S/W393R mutant genotype. Initially, she showed only moderate thrombocytopenia (platelet count 87×10^9^/l) and bruising, but did not show any symptoms of bone manifestations, and did not wish to receive treatment.

In late 2008 she agreed to commence ERT with imiglucerase 30IU/kg every 2 weeks due to worsening fatigue and the appearance of bone pain (without osteonecrosis). Thrombocytopenia was corrected within 6 months, and her splenomegaly resolved. However, after 3 years on imiglucerase she began to exhibit allergic reactions during injections (overt dyspnea with desaturation in a few seconds), which occurred despite premedication and slowing of the imiglucerase infusion rate. Immunoglobulin E anti-imiglucerase assays were conducted but did not provide interpretable findings for technical reasons. ERT was therefore discontinued and miglustat therapy was commenced with dose escalation (as described for Patient 1) and a low-lactose, low-carbohydrate diet.

At commencement of miglustat therapy in mid-2011, her platelet count and hemoglobin concentration were 168×10^9^/l and 14.9g/dl, respectively; her spleen size was 11cm. All three parameters remained stable throughout 3 years of miglustat treatment (136×10^9^/l, 14.7g/dl and 10.5cm, respectively, at last follow up), and plasma chitotriosidase remained low (439nmol/ml/hour at last follow up; Fig. 1c). No bone manifestations were apparent at start of miglustat therapy, but bone densitometry indicated osteoporosis; her lumbar spine and femur T-scores were –2.8 and –1.9, respectively. Bone MRI findings remained stable during follow up.

She lost weight (approximately 7kg) during the initial 12 months of miglustat therapy and experienced mild diarrhea and regular flatulence during the first 24 months. She had regained 2kg in body weight and showed improved tolerability to miglustat at last follow up. A fine tremor affecting her extremities was observed during the initial months of therapy, but had disappeared after 14 months of continued treatment.

## Discussion

This case series provides a real-world insight into the types of therapeutic response that can be encountered among adult patients with GD1 who have been switched from long-term ERT to miglustat. In clinical practice settings patients can be switched from ERT to SRT at their own request or based on physician recommendations for a number of reasons. In our presented cases, reasons for switching were unwillingness to continue intravenous therapy (Patient 1), unavailability of imiglucerase (Patient 2), and the occurrence of immune reactions to intravenous therapy (Patient 3).

Previous clinical trial data have shown that miglustat can maintain clinical stability in some, but not in all patients previously stabilized on ERT [11, 12]. All three of our patients showed improvements in key hematological and visceral disease parameters during initial 3 to 6 years of ERT. All three patients showed maintenance of platelets, hemoglobin and plasma chitotriosidase during a period of 4 to 8 years of subsequent miglustat therapy. Overall, these patients showed few GD1-related bone manifestations. Patient 2 developed osteopenia during 6 years of ERT, but showed no new bone manifestations or any significant progressive decrease in BMD during 2 years of subsequent miglustat therapy. Similarly, lumbar spine and femur BMD T-scores indicated osteoporosis in Patient 3 after 3 years of ERT, but bone status appeared stable during 3 years of miglustat therapy.

Two patients in this case series experienced diarrhea and/or flatulence during the initial months of miglustat therapy. However, gastrointestinal tolerability was generally good due to gradual dose escalation coupled with dietary modifications, as has been reported previously [14]. Two patients showed initial decreases in body weight, with regains noted at later follow up. Mild-to-moderate reductions in body weight are a known side effect of miglustat therapy, and should be managed with regular monitoring and dietary changes [14, 15].

As in two of the three patients presented here, mild, transient peripheral tremor has been observed during the initial months of miglustat therapy in previous clinical trials, but usually it did not impair day-to-day function and resolved during continued treatment [15]. We chose to discontinue miglustat in Patient 1 as a safety precaution following his development of peripheral neuropathy, but it was difficult to ascertain the cause of peripheral neuropathy in this patient as he had diabetes. To date, his pains have stabilized since miglustat therapy was stopped, but it is too early to evaluate whether there was any relationship to miglustat. This case demonstrates the importance of appropriate neurological assessment, including EMG, prior to starting treatment with miglustat [15].

The recent approval of another small-molecular oral SRT, eliglustat, for treating GD opens up the possibility for greater flexibility in our treatment approach. It is expected that time will tell as to whether the issues encountered in this case series will be met in future years.

## Conclusions

Overall these reports demonstrate that miglustat can maintain or even improve hematological, visceral and/or bone parameters in patients previously treated with imiglucerase, but that treatment responses can be variable. Notably, none of our patients had to stop treatment because of gastrointestinal complications. Data accrued since its approval in 2002 indicate that miglustat is an appropriate therapeutic choice in some patients, but to date there are no data to enable identification of those most likely to benefit from this SRT. The recent approval of eliglustat in August 2014 will allow further therapeutic choice, and future studies, particularly those based on real-world patient observations, could help to better define the role of these SRTs with a view to providing personalized disease management.

## Consent

All procedures followed were in accordance with the ethical standards of the responsible committee on human experimentation (institutional and national) and with the Declaration of Helsinki of 1975, as revised in 2000 (5). Written informed consent was obtained from the patients for publication of this case series and any accompanying images. Copies of the written consents are available for review by the Editor-in-Chief of this journal.

## References

[1] Cox TM, Schofield JP. Gaucher’s disease: clinical features and natural history. Baillieres Clin Haematol. 1997;10:657–89. 10.1016/s0950-3536(97)80033-9 9497857

[2] Charrow J, Andersson HC, Kaplan P, Kolodny EH, Mistry P, Pastores G, et al. The Gaucher registry: demographics and disease characteristics of 1698 patients with Gaucher disease. Arch Intern Med. 2000;160:2835–43. 10.1001/archinte.160.18.2835 11025794

[3] Elstein D, Zimran A. Review of the safety and efficacy of imiglucerase treatment of Gaucher disease. Biologics. 2009;3:407–17. 10.2147/btt.2009.3497 PMC2747339 19774208

[4] Andersson H, Kaplan P, Kacena K, Yee J. Eight-year clinical outcomes of long-term enzyme replacement therapy for 884 children with Gaucher disease type 1. Pediatrics. 2008;122:1182–90. 10.1542/peds.2007-2144 19047232

[5] Cox T, Lachmann R, Hollak C, Aerts J, van Weely S, Hrebicek M, et al. Novel oral treatment of Gaucher’s disease with N-butyldeoxynojirimycin (OGT 918) to decrease substrate biosynthesis. Lancet. 2000;355:1481–5. 10.1016/S0140-6736(00)02161-9 10801168

[6] Heitner R, Elstein D, Aerts J, Weely S, Zimran A. Low-dose N-butyldeoxynojirimycin (OGT 918) for type I Gaucher disease. Blood Cells Mol Dis. 2002;28:127–33. 10.1006/bcmd.2002.0497 12064906

[7] Elstein D, Hollak C, Aerts JM, van Weely S, Maas M, Cox TM, et al. Sustained therapeutic effects of oral miglustat (Zavesca, N-butyldeoxynojirimycin, OGT 918) in type I Gaucher disease. J Inherit Metab Dis. 2004;27:757–66. 10.1023/B:BOLI.0000045756.54006.17 15505381

[8] Pastores GM, Elstein D, Hrebicek M, Zimran A. Effect of miglustat on bone disease in adults with type 1 Gaucher disease: a pooled analysis of three multinational, open-label studies. Clin Ther. 2007;29:1645–54. 10.1016/j.clinthera.2007.08.006 17919546

[9] Hollak CE, Vom Dahl S, Aerts JM, Belmatoug N, Bembi B, Cohen Y, et al. Force majeure: therapeutic measures in response to restricted supply of imiglucerase (Cerezyme) for patients with Gaucher disease. Blood Cells Mol Dis. 2010;44:41–7. 10.1016/j.bcmd.2009.09.006 19804996

[10] Giraldo P, Alfonso P, Atutxa K, Fernandez-Galan MA, Barez A, Franco R, et al. Real-world clinical experience with long-term miglustat maintenance therapy in type 1 Gaucher disease: the ZAGAL project. Haematologica. 2009;94:1771–5. 10.3324/haematol.2009.008078 PMC2791929 19608672

[11] Cox TM, Amato D, Hollak CE, Luzy C, Silkey M, Giorgino R, et al. Evaluation of miglustat as maintenance therapy after enzyme therapy in adults with stable type 1 Gaucher disease: a prospective, open-label non-inferiority study. Orphanet J Rare Dis. 2012;7:102. 10.1186/1750-1172-7-102 PMC3552937 23270487

[12] Kuter DJ, Mehta A, Hollak CE, Giraldo P, Hughes D, Belmatoug N, et al. Miglustat therapy in type 1 Gaucher disease: clinical and safety outcomes in a multicenter retrospective cohort study. Blood Cells Mol Dis. 2013;51:116–24. 10.1016/j.bcmd.2013.04.005 23683771

[13] Biegstraaten M, Mengel E, Marodi L, Petakov M, Niederau C, Giraldo P, et al. Peripheral neuropathy in adult type 1 Gaucher disease: a 2-year prospective observational study. Brain. 2010;133:2909–19. 10.1093/brain/awq198 20693542

[14] Belmatoug N, Burlina A, Giraldo P, Hendriksz CJ, Kuter DJ, Mengel E, et al. Gastrointestinal disturbances and their management in miglustat-treated patients. J Inherit Metab Dis. 2011;34:991–1001. 10.1007/s10545-011-9368-7 21779792

[15] Miglustat (Zavesca) Summary of Product Characteristics (EudraPharm). [http://www.eudrapharm.eu/eudrapharm/productDetailsAction.do]

